# Two Perforators Improve the Extent and Reliability of Paraumbilical Flaps for Upper Limb Reconstruction

**DOI:** 10.29252/wjps.9.2.206

**Published:** 2020-05

**Authors:** Ferdinand Nangole, Alex Okello, Dorsi Jowi

**Affiliations:** Department of Surgery, University of Nairobi, Nairobi

**Keywords:** Perforator, Paraumbilical flap, Forearm, Limb, Reconstruction

## Abstract

**BACKGROUND:**

Complex defects of the forearm and arm are best reconstructed with free flaps. Free flaps are however not universally available. They require long operative time and may be contraindicated in patients with extensive injuries due to a lack of good recipient vessels. The alternatives to free flaps are distant flaps such as groin flaps, random abdominal flaps, thoracoepigastric flaps and paraumbilical perforator flaps. These are axial flaps that are limited by the angiosomes supplied by a given perforator or blood vessel. To improve the extent and reliabilities of the paraumbilical flaps, we incorporated two perforators in the flap.

**METHODS:**

A total of 17 patients with extensive forearm defects were managed by two vessel paraumbilical perforator flaps between January 2013 and December 2018. The perforators were identified by a hand-held Doppler and the flap was fashioned with the perforators at the base.

**RESULTS:**

The mean length of the flap raised was 19.5 cm and width was 8.3 cm. The median age was 39 years. All the flaps were successful with no incidence of flap necrosis and no dehiscence.

**CONCLUSION:**

Two vessel perforator flaps improved the reliability of the paraumbilical perforator flap, allowing for a bigger flap to be harvested and thus ensuring a cover of larger defects. The flaps were easy to raise and were easily tolerated by the patients.

## INTRODUCTION

Reconstruction of the upper limb defects may require flaps. Smaller defects can be reconstructed with local or regional flaps^1^ and extensive defects may however, require large flaps.^1^^,^^2^ The best option could be free flaps, since they allow early mobilization of the limb compared to distant flaps.^3^^,^^4^ However, free flaps may not be available or be contraindicated in instances, where there is suspected trauma to the recipient vessels. Paraumbilical flaps have been documented in literature as reliable and easy flaps to be used in reconstruction of upper limb defects.^5^^-^^7^

However, the flap is limited in size by the extent of blood supply. The reported safe dimensions of the flap varies from author to author with an average of about 6 cm in width to 14 cm in length.^5^^-^^7^ To further improve the reliability and the size, two perforators were incorporated into the flap instead of the traditional one perforator. We reported our experience of using two-vessel perforator flaps to cover extensive upper limb wounds. 

## MATERIALS AND METHODS

**Fig. 1:**
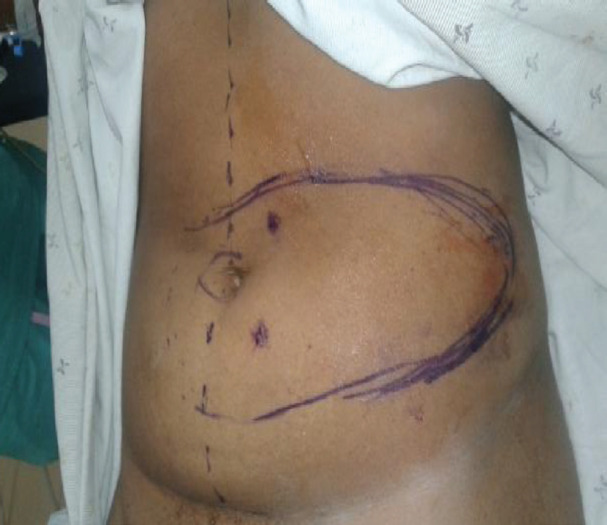
Two perforators identified about 3 cm from the umbilicus at the base of the flap

This study was a prospective review of patients with extensive wounds of the upper limb operated with two-vessel perforator paraumbilical flaps in the period between January 2013 and Dec 2018. The study was approved by the local ethical and research committee. Consent or assent of the study was sought from the patient. Once the wound was ready for closure with the use of a hand-held Doppler, two ipsilateral paraumbilical perforators were identified (Figure 1). 

**Fig. 2:**
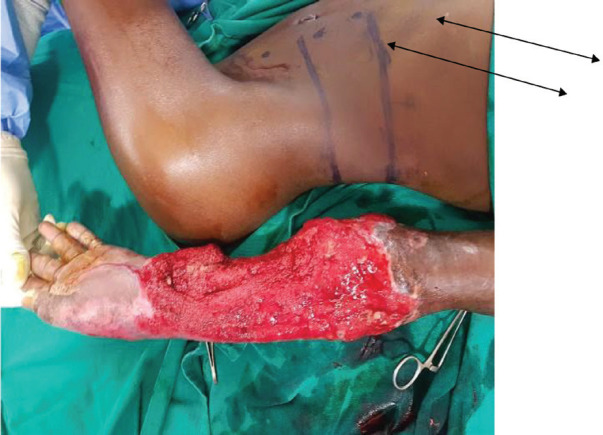
Patient with extensive arm defect that required free nerve grafts to reconstruct both the median and ulnar nerve with two perforators of paraumbilical flap planned to cover the wounds. The flap dimensions were determined by the size of the wound to be covered. Note the two perforators marked by arrows

**Fig. 3:**
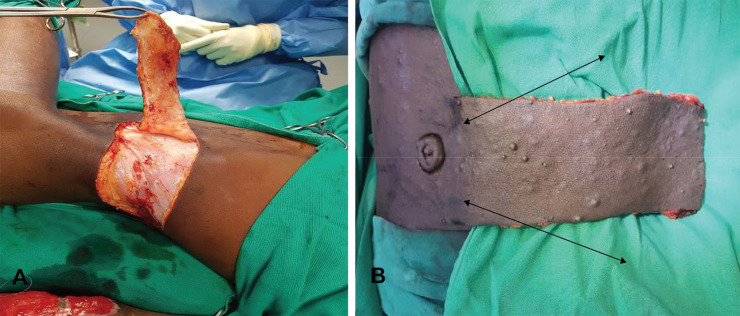
**A.** Two vessel perforator flaps raised in a subfascial plane from distal to proximal, with the donor site primarily closed. **B. **Paraumbilical flap raised: The donor site was closed primarily. Note the arrows pointing at the 2 perforators

**Fig. 4:**
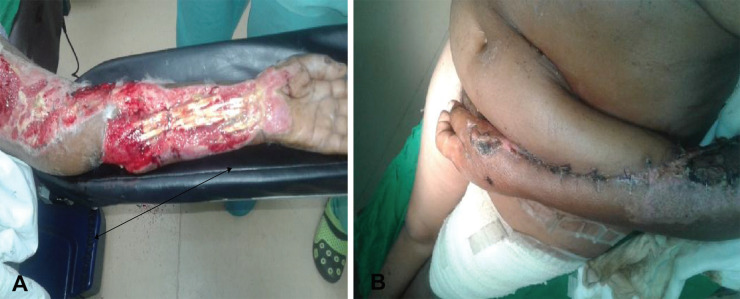
**A.** Patients with exposed tendons and neurovascular structures ready to be covered with paraumbilical flap of dimensions 24×10 cm. **B.** Perforator paraumbilical flap successfully anchored to the recipient site

**Fig. 5:**
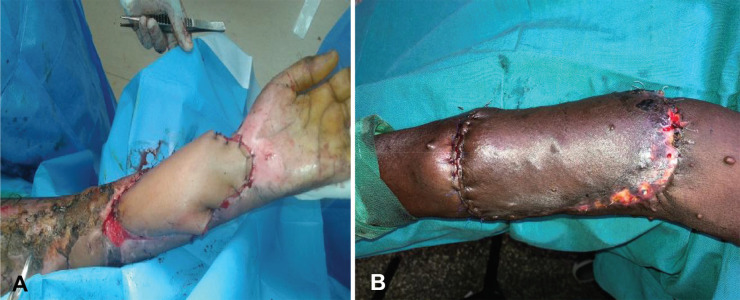
**A.** Left volar arm defect fully covered with the two vessel paraumbilical flap immediately after separation. **B.** Left arm wound fully covered with the paraumbilical flap at 2 months of follow up. Note that the defects had extended between the wrist and the elbow

The flap was fashioned around the perforators after determining the length and width based on the size of the defect to be closed (Figure 2). The flap was raised from the distal to the proximal end in the subfascial plane until the perforators were reached (Figure 3). The flap was then advanced into the defect and secured with sutures (Figure 4). After 21 days, the flap was detached and the donor wound closed primarily (Figure 5). The variables measured were the size of the flap, flap-related complications and donor site morbidity.

## RESULTS

Wounds of the arm in a total of 17 patients were closed by two vessels perforator paraumbilical flaps. The age of patients ranged from 6 to 65 years, and the mean age was 35 years. Seven patients had defects involving the hand. Another seven patients had defects involving the forearm and three had defects both in the hand and the forearm. Nine patients had injuries secondary to the road traffic accidents, three assault, two burns, three infective courses and one post-tumor surgery. 

The smallest flap utilized in the study was 14×7 cm and the largest flap was 30×10 cm. The mean duration taken for the flap to be detached was 22.6 days. The mean flap surface area utilized was 164 cm^2^. The flap donor site was closed primarily in all cases. All the flaps survived with no incidence of flap necrosis, dehiscence or infection. The donor sites healed well with no sepsis or dehiscence of the wounds either. Hypertrophic scars were noted in four patients at six months of follow-up. The patients’ characteristics, aetiology and the size of the flaps utilized to cover the defect were demonstrated in Table 1. 

**Table 1:** The patients’ characteristics, aetiology and the size of the flaps utilized to cover the defect

**Age (years)**	**Sex**	**Aetiology **	**Anatomical ** **location**	**Defect size (cm)**	**Length of flap (cm)**	**Width of flap (cm) **	**Flap surface area (cm** ^2^ **)**
6	M	RTA	Volar forearm	20×5	22	6	132
20	F	RTA	Dorsum of the hand	18×7	20	8	116
28	M	RTA	Dorsum of the hand	12×5	14	6	84
29	M	Assault	Dorsum of the hand	14×6	16	7	112
35	F	Assault	Forearm	20×8	22	7	154
65		RTA	Volar forearm defect	28×9	30	10	300
38	F	Cellulitis	Dorsum of the hand	20×10	23	11	253
45	M	RTA	Forearm dorsum	19×12	22	7	154
32	M	Crush injury	Dorsum of the hand	15×7	17	8	136
27	F	Degloving injury hand	Dorsum and volar	22×10	24	11	264
18	F	RTA, motor bike	Dorsum forearm	23×9	24	10	240
17	F	Assault, arm	Elbow joint injury	10×7	12	6	72
60	M	Burn wounds forearm	Dorsum of the forearm	16×9	17	10	170
48	M	Fuorniers gangrene	Dorsum hand and forearm	18×10	19	11	209
65	F	Cellulitis	Forearm and dorsum	17×8	17	10	170
45	M	Tumour	Forearm/Elbow	19×12	21	13	273
18	M	Electrical burns	Forearm	10×6	12	7	84

RTA: Road traffic accidents, M: Male, F: Female

**Fig. 6:**
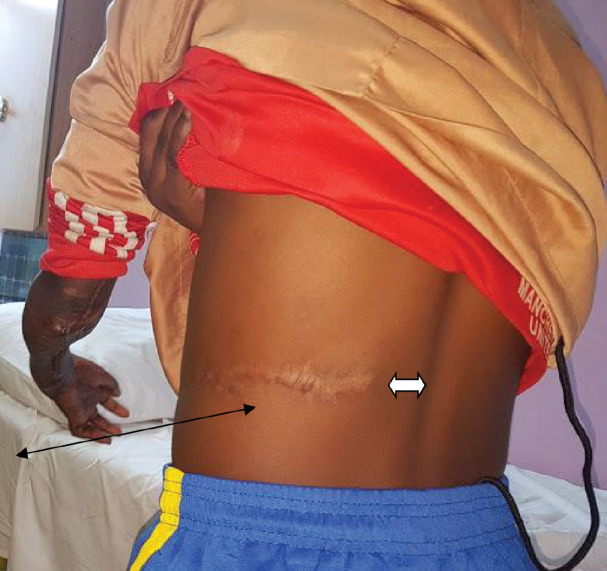
Note the scar on one of the patients who had paraumbilical perforator flaps. The flap extended to just about 4 cm from the spinal cord

More than half the flaps extended to the posterior axillary line with some extending to about 4 cm from the spinal column (Figure 6). With increased vascularity, the safety margins of the flaps were extended from the reported mid axillary line to beyond the posterior axillary line. With this, we were able to harvest large flaps that enabled us to cover larger defects that otherwise could only be covered by free flaps.

## DISCUSSION

Upper limb reconstruction demands good functional outcome. Wounds with exposed bones, tendons or neurovascular structures should be reconstructed with flaps. Among commonly used flaps are the groin flaps, abdominal flaps, free tissue transfers and paraumbilical flaps.^1^^,^^2^ Groin flaps have been the workhorse flap for reconstruction of defects of the hand since its inception in 1972 by Mcgregor.^8^ The flap is raised as an axial flap based on the superficial circumflex iliac artery. It has an excellent donor site that is not visible. However, the size of the flap is limited and largely limited to small or medium defects of the hand and not large defects as encountered in our series.^9^

Another flap commonly used is the bipedicle abdominal flap.^1^^,^^2^ This flap has the advantage of being an easy flap to raise. Being a random flap, it is limited by the size and is thus only has small defects. Free flaps are probably the gold standard in managing extensive tissue loss of the upper limb.^3^^,^^4^ Some of the commonly used free flaps are the Parascapular flaps, the anterior lateral thigh flaps and the Lattismus dorsi muscle flaps.^3^^,^^4^

With free flaps, both the soft tissue and functional reconstruction can be achieved. The disadvantages of the free flap, however, include a long learning cover, long operative times and a demanding flap monitoring period. Further still, a good proportion of the injuries may involve injuries to the recipient vessels, making it hard to utilize such vessels. Even further, free flap surgeries are not universally available at many centers in the middle and developing countries without such services.^10^

Paraumbilical perforator flaps are raised on the perforators of the deep inferior epigastric vessels. The perforator is located two to three centimetres lateral to the umbilicus.^5^^,^^6^ The safety dimensions of this flap, when raised on a single perforator has not been conclusively decided, but in literature, it seems to vary from author to author. YImuz *et al.* in a series of eleven patients demonstrated a flap with a maximum size of 5 cm to 14 cm.^11^ Jim Wang et al. in a series of 14 patients reported a flap of mean dimensions of 6 cm to 8 cm in width and 16 cm to 20 cm in length.^12^

In a series of 12 patients, flap dimensions ranging from 6 cm to a maximum length of about 18 cm were used.^13^ Most of his flaps extended up to the anterior axillary line, with only five extending to the mid axillary line.^7^ The overall flap survival was about 75%, with the rest either having total or partial flap necrosis. In our series, all flaps had two perforators identified within 3 cm from the umbilicus. The mean flap dimension was 19.5 cm in length and had a width of 9 cm. Our flaps ranged from 12 cm to 30 cm. More than half the flaps extended to the posterior axillary line with some extending to about 4 cm from the spinal column (Figure 6).

The mean flap surface area was 164 cm^2^. There was no incidence of flap necrosis in any of the patients we operated on. The only reason that could be attributed to the good flap survival and extensive flap length in our series when compared to the previous studies, is the fact that we had incorporated two perforators and thus essentially supercharged the flaps. The two vessels were able to provide a rich arterial and venous drainage that were able to maintain the vascularity and increase the angiosome zones of the flaps.

With increased vascularity, the safety margins of the flaps were extended from the reported mid axillary line to beyond the posterior axillary line. With this, we were able to harvest large flaps that enabled us to cover larger defects that otherwise could only be covered by free flaps. The two-vessel perforator flaps allow for an enhanced vascularity of the paraumbilical flap, which in turn allows one to extent the limits of the flap dissection, almost up till the spinal column. This allows for an extensive flap that could cover a wider range of forearm defects with good surgical outcomes, thus obviating the need for free flaps in some cases. The flaps are also more reliable with better flap take rates.

## CONFLICT OF INTEREST

The authors declare no conflict of interest.
